# SUPPORTING AGING THROUGH HOUSING CHOICE: A DISCUSSION TOOL FOR COLLABORATION TOWARD LOCAL SOLUTIONS

**DOI:** 10.1093/geroni/igae098.4298

**Published:** 2024-12-31

**Authors:** Nancy Berlinger

**Affiliations:** The Hastings Center, Garrison, New York, United States

## Abstract

This abstract concerns applied research and new open-access publications that will include piloting data from late September 2024, hence its submission as late-breaking research. This paper will present a discussion tool to support local discussion and collaboration to meet the housing needs of moderate-income older Americans. It has been informed by insights from national and regional aging and policy audiences and reflects findings from a mixed-methods study presented at GSA in 2022 concerning pandemic-era innovations that held promise for policy and practice concerning housing equity for older adults. Research shows that the vast “missing middle” population of older adults who do not qualify for housing subsidies and cannot afford market solutions geared to wealthy individuals struggles to pay for both housing and care. Within the national shortage of affordable housing, there is a shortage of housing that meets the needs of this population: housing that is affordable, accessible or adaptable, and a good fit for an aging person’s needs. This paper will share a slide-deck format tool and other resources that offer ways to explain housing needs, clarify realistic options, anticipate challenges, analyze and share success stories, and encourage productive collaboration among local policymakers, community members, housing developers, service providers, and other participants.

